# The complete mitochondrial genome and phylogenetic analysis of the Hetian chicken (Gallus gallus)

**DOI:** 10.1080/23802359.2020.1791020

**Published:** 2020-07-15

**Authors:** Jingjing Gu, Sheng Li

**Affiliations:** a College of Animal Science and Technology, Hunan Agricultural University, Changsha, China; b Hunan Key Laboratory for Genetic Improvement of Animals, Changsha, China; c Hunan Engineering Research Center of Poultry Production Safety, Changsha, China; d Maxun Biotechnology Institute, Changsha, China

**Keywords:** Hetian chicken, mitochondrial genome, next generation sequencing

## Abstract

In this study, the complete mitochondrial genome of Hetian chicken (Gallus gallus) was obtained by using next generation sequencing method. The total length of complete mitogenome sequence is 16,784 bp, containing one control region, 2 ribosomal RNAs, 13 protein-coding genes and 22 transfer RNA genes. This work provides a valuable source of data for the study of the evolution of Gallus gallus mitochondrial genome and contributes to Hetian chicken breeding improvement program.

Hetian chicken (Gallus gallus) is one of the important local chicken breeds in China. It is a slower-growing broiler with excellent meat quality. Its typical physical appearance is characterized by ‘three yellow,’ ‘three black’ and ‘trigeminal comb.’ The skin and shins of Hetian chicken are yellow, the basic color of beak is brown, the tip of beak is light yellow, the tail feather and sickle feather are shiny black, the main wing feather is black inlaid with golden edge, and the comb is a single comb with upright rear natural bifurcation. To understand the genetic background of this breed, we reported the complete sequence of the mitochondrial genome of Hetian chicken for the first time. The chicken sample used in this study was collected in Changting County (25.83 N and 116.36 E), Fujian Province, China. The muscle specimen (Voucher No. HT150752) was stored at −80 °C in the Museum of Hunan provincial key laboratory for genetic improvement of domestic animal, Changsha, China. The total genomic DNA was extracted from this muscle specimen and used as input materials to build sequencing libraries. The sequencing libraries were then sequenced on Illumina Hiseq 2500 high-throughput sequencing platform. In total, we generated 10.42 Gb raw data and this sequence has been deposited in the NCBI Sequence Read Archive (SRA) with accession number SRR4302052. The assembled mitochondrial genome sequence was deposited in GenBank with accession number MT555048.

We analyzed the mitochondrial genome sequence of Hetian chicken using the total length of 16,784 bp. The mitogenome was annotated by tRNAscan-SE 2.0 (Chan and Lowe 2019) and MITOS (Bernt et al. 2013). The overall base composition of the complete mitogenome is 30.2% A, 23.7% T, 32.5% C, and 13.5% G and slightly biased toward A + T nucleotides (53.9%). It contains the typical vertebrate mitochondrial structure, including 1 noncoding control region (D-loop region), 13 protein-coding genes (PCGs), 2 ribosomal RNA genes (rRNAs) and 22 transfer RNA genes (tRNAs). All these genes have 10 overlaps in the length of 1–10 bp. One protein-coding gene (*ND6*) and 8 tRNA genes are encoded on the light (L) strand. However, the other 2 rRNA genes, 12 protein-coding genes and 14 tRNA genes are encoded on the heavy (H) strand. The initiation codon of most PCGs is ATG except for *COX1* being GTG. There are four types of termination codon for PCGs, including TAA, TAG, AGG, and an incomplete termination codon ‘T–,’ which is the 5′ terminal of adjacent gene (Anderson et al. 1981). Among 13 PCGs, *ND5* (1818 bp) is the longest while *ATP8* (165 bp) is the shortest PCG. The 22 tRNA genes are distributed among rRNA and PCGs, ranging from 66 to 75 bp in length. The lengths of 12S rRNA and 16S rRNA genes are 977 bp and 1572 bp, respectively.

The phylogenetic position of Hetian chicken is revealed by constructing the neighbor-joining (NJ) phylogenetic tree. We retrieved thirty-seven diversified chicken breeds to build the NJ tree with 1000 bootstrap replicates using Mega 7.0 (Kumar et al. 2016). The results (Figure 1) shown the Hetian chicken and some chicken breeds grouped to one clade which indicates that Hetian chicken has the closest maternal relationship with Daweishan Mini, Nandan and Henan autochthonic. However, Hetian chicken has the farthest genetic distance with the Zhuxiang. This work provides a valuable source of data for the study of the evolution of Gallus gallus mitochondrial genome and contributes to Hetian chicken breeding improvement program.

**Figure 1.**
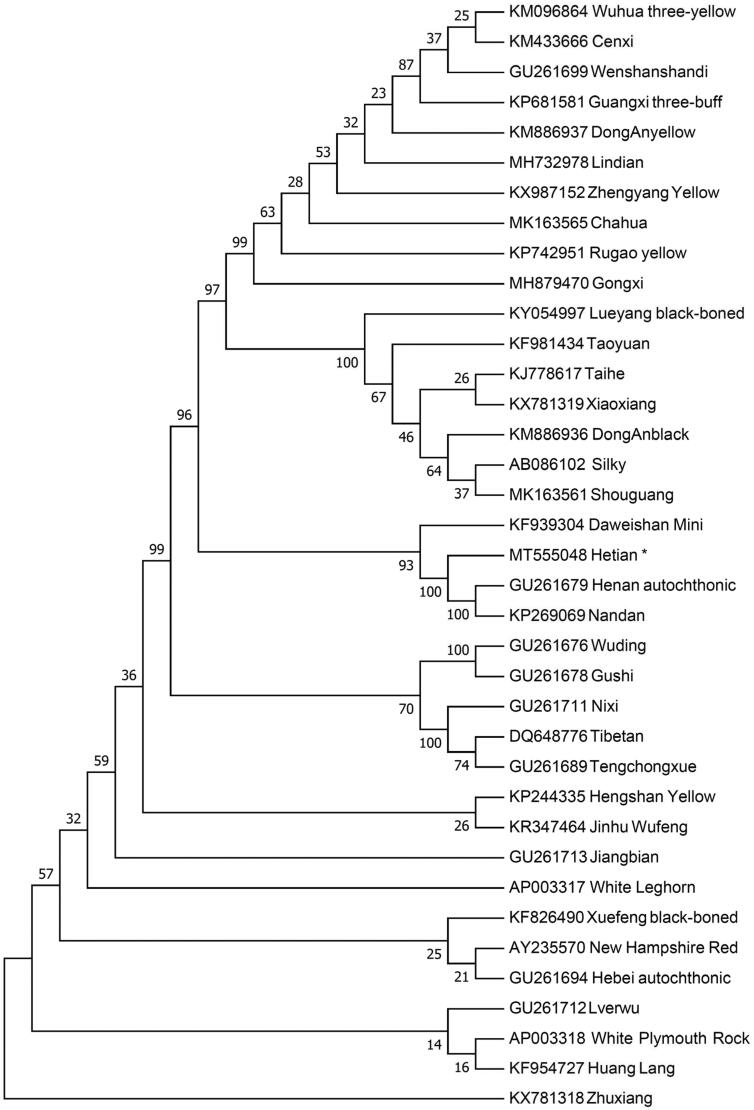
Neighbor-joining tree based on the complete mitochondrial DNA sequence of 37 chicken breeds. GenBank accession numbers are given before the species name.

## Data Availability

The sequence data that support the findings of this study are openly available in the NCBI Sequence Read Archive (SRA) at http://www.ncbi.nlm.nih.gov/sra/ with accession number SRR4302052. The complete mitochondrial genome of Hetian chicken (Gallus gallus) is openly available in GenBank at http://www.ncbi.nlm.nih.gov/genbank with accession number MT555048.

## References

[CIT0001] Anderson S, Bankier AT, Barrell BG, de Bruijn MH, Coulson AR, Drouin J, Eperon IC, Nierlich DP, Roe BA, Sanger F, et al. 1981. Sequence and organization of the human mitochondrial genome. Nature. 290(5806):457–464. 7219534 10.1038/290457a0

[CIT0002] Bernt M, Donath A, Juhling F, Externbrink F, Florentz C, Fritzsch G, Putz J, Middendorf M, Stadler PF. 2013. MITOS: improved de novo metazoan mitochondrial genome annotation. Mol Phylogenet Evol. 69(2):313–319. 22982435 10.1016/j.ympev.2012.08.023

[CIT0003] Chan P, Lowe T. 2019. tRNAscan-SE: searching for tRNA genes in genomic sequences. Methods Mol Biol. 1962:1–14. 31020551 10.1007/978-1-4939-9173-0_1 PMC6768409

[CIT0004] Kumar S, Stecher G, Tamura K. 2016. MEGA7: Molecular Evolutionary Genetics Analysis version 7.0 for bigger datasets. Mol Biol Evol. 33(7):1870–1874. 27004904 10.1093/molbev/msw054 PMC8210823

